# Changes in Functional Compounds, Volatiles, and Antioxidant Properties of Culinary Herb Coriander Leaves (*Coriandrum sativum*) Stored Under Red and Blue LED Light for Different Storage Times

**DOI:** 10.3389/fnut.2022.856484

**Published:** 2022-05-12

**Authors:** Tinotenda Shoko, Vimbainashe E. Manhivi, Malebo Mtlhako, Dharini Sivakumar

**Affiliations:** Phytochemical Food Network Group, Department of Crop Sciences, Tshwane University of Technology, Pretoria, South Africa

**Keywords:** light treatment, volatile compounds, antioxidant activities, phenolic compounds, β-carotene

## Abstract

This study evaluated the influence of red (630–640 nm) and blue (450 nm) light-emitting diodes (LED) lights on the changes in antioxidant constituents, activity, volatile compounds, and overall acceptability of Coriander leaves (*Coriandrum sativum*) during post-harvest storage. Coriander leaves are harvested at commercial maturity, packed in polyethylene terephthalate punnets, and exposed for 2 h to the red LED or blue LED lights separately during storage at 5°C and 85% RH up to 9 days. Coriander leaves exposed to the white light (2 h) and continuous darkness served as controls. Samples were removed from cold storage at 3, 6, and 9 days to determine the antioxidant constituents, their activity, retention of volatile compounds, and overall acceptance. Coriander leaves exposed to red and blue LED lights for 2 h showed a commercially allowable mass loss of up to 9 days compared to the other treatments. Compared to those exposed to red LED light (2 h) and the control, leaves exposed to blue LED light for 2 h and stored for 3–9 days showed a reduction in colour change (Δ*E*). The β-carotene content significantly peaked at 44.55% on day 6 in coriander leaves exposed to the red LED light. However, leaves exposed to blue and red LED light showed an increase in total phenolic content by 9.34 and 6.39% on day 9, respectively. Exposure to blue LED lights increased the antioxidant activities (DPPH, ABTS, and FRAP), quercetin content, and the concentration of typical coriander aroma, 2-tridecenal, 2-dodecenal, (*E*), and *Z*-9-19 hexadecenal on day 9. Coriander leaves exposed to blue LED light (2 h) and stored up to day 9 scored a higher acceptance level by the panellists. Thus, blue light LED treatment during post-harvest storage can be recommended to retain the antioxidant property of coriander leaves.

## Introduction

Coriander (*Coriandrum sativum*), belonging to the family Umbelliferae, leaves are widely used in culinary as a flavouring agent and are well-known for their medicinal properties. The origin of coriander is from the Mediterranean region. Coriander leaves are also a rich source of vitamins (vitamin B2: 60 mg 100 g^−1^, vitamin C: 135 mg 100 g; vitamin A: 10,460 I.U (International unit) 100 g^−1^) (1). Phenolic compounds, such as chlorogenic acid, vanillic acid, caffeic acid, ferulic acid, p-coumaric acid, quercetin, kaempferol, and apigenin are reported in coriander leaves (1, 2). The leaves also contain carotenoids, luteolin, β-carotene, and have a distinct aroma (1). The leaves are widely used to flavour food or mask unpleasant odours in certain foods. The aroma of coriander leaves plays a vital role in determining the value of the product. The (*E*)-2-decenal, dodecenal, (*E*)-2-tridecenal, and dodecanal are major volatile compounds present in the leaves (3). It is proven that the phenolic compounds show antioxidant activity, thus, contributing towards protective effects *in vivo* to benefit the consumers (3). Currently, consumers are more interested in consuming healthy foods that are rich in phytochemicals or bioactive compounds. However, a rapid decline in coriander leaf quality as a result of post-harvest senescence causes significant losses for the industry. The shelf life of coriander leaves is very short, even when refrigerated. This has resulted in a marked increase in price and scarcity of the product. Leaf yellowing, shrivelling, and chilling injury affect the quality of coriander leaves during post-harvest storage. Hassana and Mahfouz (4) concluded that the 1-MCP treatment prolonged the shelf life of coriander leaves significantly compared to an untreated control with minimal mass loss at a storage temperature of 5 or 15°C. According to O'Hare and Wong (5), 1-MCP probably does not provide sufficient or consistent benefits to justify the costs involved for these vegetables (5). Light-emitting diodes (LEDs) in the production of leafy vegetables and herbs are becoming popular globally to increase yield and product quality due to their desirable properties. As a non-chemical sanitisation method, it is also considered a green alternative to minimise post-harvest losses during storage and transportation. It has been shown that LED light irradiation extends the shelf-life and improves the nutraceutical quality of lettuce and broccoli (6, 7), and is emerging as a common, effective treatment for harvested vegetables. Furthermore, consumers are more interested in consuming healthy foods that contain phytochemicals or bioactive compounds.

Light quality is one of the main factors of the plant environment. It acts through specific photoreceptors sensitive to various wavelengths (phytochromes, cryptochromes, phototropin, and UVR8), thus, directly offering physiological and chemical processes in plants (8). Light-emitting diodes (LED) are becoming popular in horticulture due to their desirable wavelengths, low thermal energy, non-hazardous (no mercury) effects, long life expectancies and affordability energy efficiency, and non-chemical sanitisation treatment that can be used as an alternative green technology to minimise post-harvest losses during storage and transportation (9).

Red sweet pepper fresh cuts exposed to the red LED light maintained the highest concentrations of β carotene, chlorophyll, and lycopene in yellow, green, for 7 days (10). Furthermore, in yellow and green fresh-cut sweet peppers, red LED light and in red fresh-cut sweet pepper, blue LED light enabled the accumulation of phenolic compounds by inducing the activity of phenylalanine ammonia-lyase (PAL) (10). In avocados, red LED lights improved the accumulation of epicatechin content in the fruit by upregulating the PAL genes (11). LED red light treatment improved the lycopene and β-carotene, total phenols, and flavonoids in tomatoes during post-harvest storage (10). Conversely, blue LED treatment improved the lycopene, chlorogenic acid, caffeic acid, and rutin content after 7 days of storage (11). Moreover, the red or blue LED lights improved the antioxidant properties of fresh produce and are regarded as an efficient strategy to increase health-promoting compounds to add health benefits to the consumers (12). However, there is a limited information available on the influence of LED quality on the changes in different phenolic compounds and aroma compounds in coriander leaves after post-harvest storage. Therefore, this study was aimed to evaluate the mass loss, changes in colour properties, antioxidant components, scavenging activities, and volatile compounds in coriander leaves exposed to red or blue LED light for 2 h, stored at 5°C for up to 9 days. Finally, to select the best-LED light and storage period combinations to improve the concentration of antioxidant constituents and their activity for health benefits with retention of volatile compounds and higher overall acceptance.

## Materials and Methods

### Chemicals and Reagents

The following reagents of analytical grade were used: methanol, Folin–Ciocalteu reagent, sodium carbonate, gallic acid, sodium acetate trihydrate, sodium hydrogen phosphate dihydrate, glacial acetic acid, sodium persulphate, TPTZ (2,4,6-tripyridyl-*s*-triazine), iron chloride (III) hexahydrated, (±)-6-Hydroxy-2,5,7,8-tetrathylchromane-2-carboxylic acid (Trolox), ABTS (2,2′-azinobis (3-ethylbenzothiazoline-6-sulfonic acid), phosphate, sodium chloride, DPPH (2,2-diphenyl-1-picrylhydrazyl), formic acid, acetone, anhydrous sodium sulphate, *n*-hexane (HPLC grade), acetonitrile (HPLC grade), and Octanal (GC). All reagents were purchased from Sigma-Aldrich, Johannesburg, South Africa.

### Plant Material

During 2019, coriander plants were grown in a closed hydroponic system with 5 L black polyethylene growing bags filled with coir-sand growing medium under a 5 m high, 12 m long, 12 m wide tunnel covered with white shade nets with 40% shading as described by Buthelezi et al. (13) at the Qutom farms, Pretoria, South Africa (latitude: 25° 37′ S, longitude: 28° 12′ E; elevation: 1,173 m above sea level). The plants were grown strictly according to commercial practises for protected culture production, and they were irrigated daily with drip irrigation. Fertigation was performed with a soluble complete nutrient solution (Hygrotech^®^, Pretoria, South Africa).

The coriander leaves (*Coriandrum sativum* L.) were selectively harvested at 6:00 am and transported to Tshwane University within 3 h of harvest in a refrigerated vehicle at 12°C. Leaves with uniform size and an absence of visual defects were selected, and 20 g of leaves were randomly packed and non-sealed, in commercially used polyethylene terephthalate (PET) punnets (13).

### LED Treatments and Storage

**Figure 1:**
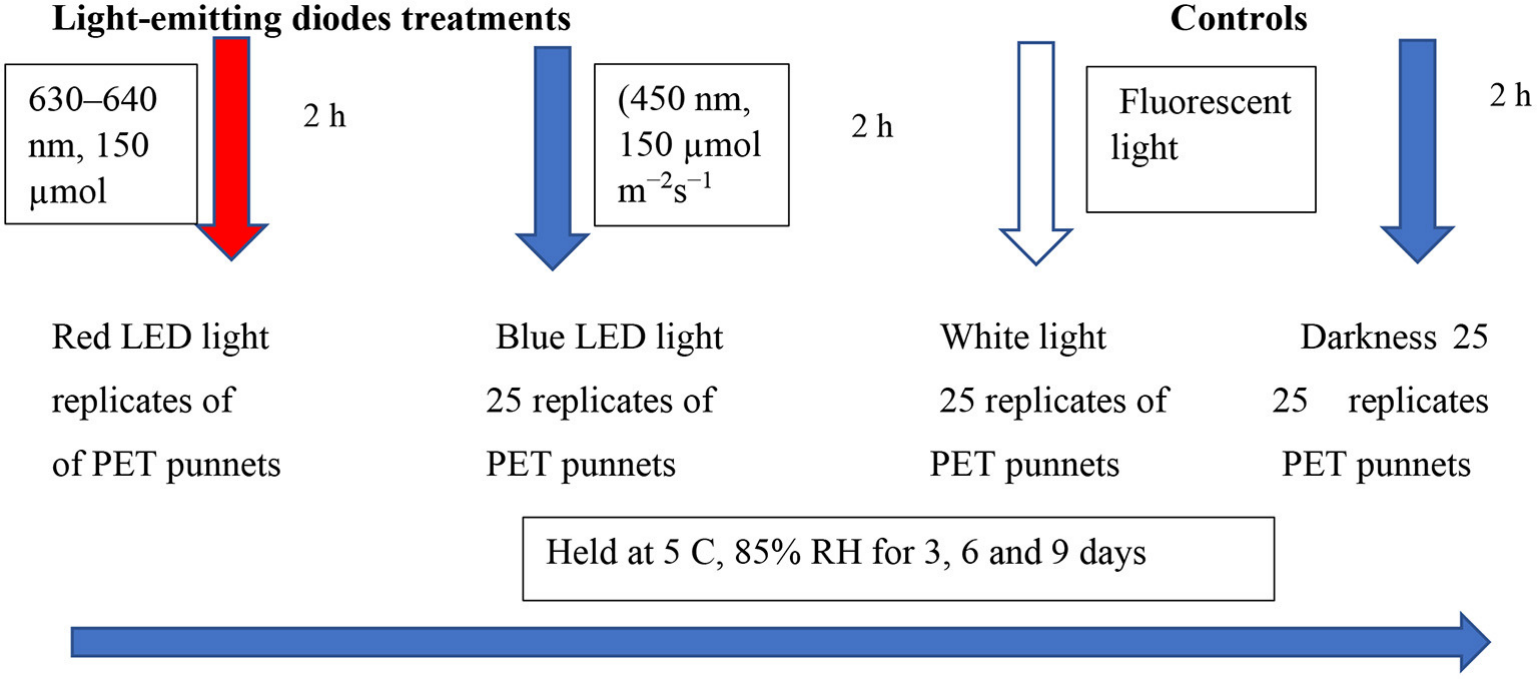
Shows the experimental layout.

Sets of 25 replicates of PET punnets were laid out randomly and exposed to the red LED light [(630–640 nm, 150 μmol m^−2^s^−1^), blue LED light], and white light (white cool fluorescent lamps; Phillips, Fluotone 40 W, Philips Africa (Pty) Ltd, Johannesburg, South Africa) for 2 h per day. The LED lights were fixed at a distance from the punnets, at ~30 cm. The white light and continuous darkness were included as controls in this study. The entire setup was stored at 5°C for 85% RH for up to 3, 6, and 9 days to simulate the cold storage at the supermarkets (Figure 1). Thereafter, samples were removed from cold storage to determine the overall marketability and biochemical analysis at designated intervals. Sets of 20 replicates (punnets) per treatment were used to determine the overall marketability, while five replicates per treatment were snap-frozen in liquid nitrogen and stored at −80°C for further biochemical analysis. Fresh leaf samples were used to investigate the influence of LED light exposure for 2 h on the changes in coriander leaf volatile compounds.

### Loss of Mass

The initial mass of 20 replicates (punnets) per post-harvest treatment was weighed in grammes (g) on day 0, before the LED storage trials. After completion of each designated storage (3, 6, and 9), period samples were removed from cold storage and a standard scale (Inscale Scales Ltd, Milton Keynes, UK) was used to record the mass. The differences in mass between the sampling days were calculated concerning the initial mass and expressed as a percentage of loss of mass.

### Colour Properties

The colour values of coriander leaves were measured using a Minolta CR-400 chromameter (Minolta, Osaka, Japan). According to the International Commission on Illumination (CIE) CIE colour system, the colour coordinate a^*^ is associated with the colour red or green if its value is positive or negative. Likewise, the colour coordinate b^*^ is described as a yellow colour when positive. Based on the following formula (14), colour changes *(*Δ*E*) were calculated. For the determination of colour changes, measurements were taken at three points on each replicate.

The total colour difference (ΔE) was calculated as shown below:


$$\Delta E=\left[\left({\Delta L}^{*}\right)2+\left({\Delta a}^{*}\right)2+\left({\Delta b}^{*}\right)2\right]$$

½ where

ΔL^*^ = Colour difference is calculated as the sample L value minus standard.

Δa^*^ = Colour difference is calculated as the sample a value minus standard.

Δb^*^ = Colour difference is calculated as the sample b value minus standard.

### Total Chlorophyll Content

Determination of the chlorophyll a (*Chl a*) and b (*Chl b*) contents were performed according to Managa et al. (15). A freeze-dried powdered leaf sample (0.02 g; three replicate samples per post-harvest treatment) was mixed with a 5 mL acetone-hexane combination (4:6 v/v) and extracted for 2 h. Following this, the sample mixture was centrifuged (Hermle Labortechnik Wehingen, Germany) at 2,500 rpm for 10 min at 4°C. The supernatant was placed in a microplate reader, and the absorbance was measured at 453, 646, and 663 nm.

The *Chl a* and *Chl b* values were calculated using the formulas below:


$$\begin{array}{l} Chl\;a\;=\;15.65\;A663\;-\;7.340\;A646\\ Chl\;b\;=\;27.05\;A646\;-\;11.21\;A663 \end{array}$$

The total chlorophyll content was expressed as mg 100 g^−1^ on a dry weight basis.

### Sensory Evaluation

Overall acceptance of the product was determined using a trained panel of 20 panellists (10 males and 10 females) who were between the age of 20–35 (8). The panellists were selected based on the frequent consumption of dishes that were garnished with coriander leaves. The assessment was made on overall acceptance by presenting randomised samples. Ten sets of 20 g (10 replicates) per treatment were displayed to the panellists on white plates. The evaluation was carried out to determine the overall acceptance based on the absence of discoloration due to yellowing, typical aroma, shrivelling, using a 10 point hedonic scale. For shrivelling and yellowing; 10–9 = very bad 50% unmarketable, 8–7 = poor 25% yellowing/shrivelling, unmarketable; 6–5 = average 10% yellowing/ shrivelling, limited marketability; 4–3 = good, and 1–5% yellowing/shrivelling, marketable; 1–2 = excellent. For typical aroma and overall acceptance; 10–9 = excellent, 8–7 = good, marketable; 6–5 = average, limited marketability; and 4–3 = poor, unmarketable.

### Extraction of Phenolic Compounds

Coriander leaf extract was made by using 200 mg of coriander leaf powder and homogenising in 80% methanol/water (v/v) and thereafter, sonicating for 30 min at 30°C. The mixture was then centrifuged (Hermle Labortechnik, Type 2326K, 2010, Wehingen, Germany) at 3,420 *g* for 15 min at 25°C. Quantification of total phenols, phenolic components, and antioxidant assays was carried out using the supernatant.

### Total Phenolic Content

Total phenolic content was determined using a method by Buthelezi et al. (13). The reaction mixture included an aliquot of 10 μL of supernatant extract, 20 μL Folin–Ciocalteu reagent, and 20 μL of 7.5% Na_2_CO_3_. The absorbance was measured at 750 nm using a microplate reader (Spectrostar Nano, BMG Labtech GmbH, Germany). The total of the phenolic compounds was calculated using a gallic acid standard curve and the results were expressed as mg 100 g^−1^ gallic acid equivalents (GAE) on a fresh mass basis.

### Antioxidant Properties

#### Ferric Reducing Antioxidant Power (FRAP)

The FRAP assay was performed according to Seke et al (16). using FRAP solution (0.3 mM sodium acetate (pH 3.6), 10 mM TPTZ, and 20 mM FeCl_3_, mixed in a ratio of 10:1:1 v/v/v, respectively). FRAP solution (950 μL) was mixed with 50 μL of the leaf extract at 37°C. The absorbance was read at 593 nm using a microplate reader (Spectrostar Nano, BMG Labtech GmbH, Germany). A calibration curve was constructed using Trolox (0.2–10 mM) and expressed as mmol Trolox equivalents antioxidant capacity (TEAC) per 100 g fresh mass (mmol TEAC 100 g^−1^).

#### 2,2′-Azino-Bis(3-Ethylbenzothiazoline-6-Sulfonic Acid) (ABTS)

The ABTS assay was performed according to Seke et al. (16). ABTS radical cation (ABTS ±) was produced by the reaction of the ABTS stock solution (7 mM), with 4.9 mM potassium persulphate at the ratio of 1:1 by incubating in the dark at 25°C for 12–16 h prior to use. An aliquot of 40 μL of leaf extract (at different concentrations) was pipetted to 200 μL of the ABTS+. The resulting reaction mixture was pipetted in a 96-well microplate reader at 37°C for 10 min and the decrease in absorbance at 734 nm was read and expressed as the IC_50_.

#### 2,2-Diphenyl-1-Picrylhydrazyl (DPPH) Radical Scavenging Activity

The DPPH activity radical scavenging activity was performed according to the method previously described by Seke et al. (16). Different concentrations of leaf extracts (30 μL) reacted with 180 μL of the DPPH solution (0.13 mg mL^−1^) for 20 min in darkness, and the absorbance was measured at 515 nm using a microplate reader (Spectrostar Nano, BMG Labtech GmbH, Germany) and the antioxidant capacity expressed as IC_50_ in μg mL^−1^.

### Quantification of Phenolic Components

The different phenolic components of coriander leaves were extracted using the hydro-methanol extracts mentioned in 1.6, and, thereafter, quantified using HPLC-DAD, Model Flexar TM 89173-556 (PerkinElmer, Waltham, MA, USA), as described previously by Seke et al. (16). Before the quantification of different phenolic components, the supernatants were filtered using PTFE membrane philtres, and 3 μL was injected into HPLC-DAD, Model Flexar TM 89173-556 (PerkinElmer, Waltham, MA, USA). The separation of different phenolic components was obtained on a Waters HSS T3 C18, 2.1 × 100 mm, 1.7 μm column. The flow rate was 0.3 mL min^−1^, and the column temperature was maintained at 55°C. The mobile phase condition was set similar to that of Seke et al. (16) [mobile phase A; 0.1% formic acid, and mobile phase B; 0.1% formic acid in acetonitrile].

The gradient initiated at 100% solvent A for 1 min and changed to 28% B for 22 min in a linear way, and, subsequently, to 40% B for 50 s and a wash step of 1.5 min at 100% B, followed by a re-equilibration to the initial conditions for 4 min. Phenolic compounds were identified at 265, 280, and 320 nm, quantified using the standards, and expressed in mg per kg of fresh mass. The limit of detection (LOD), limit of quantification (LOQ), and the calibration curve equations for the standards are given in Supplementary Table 1.

### β-Carotene Content

The β-carotene was extracted according to the method of Panfili et al. (17) using 0.25 g of samples extracted in a 5 mL mixture of acetone: hexane (1:1) with 0.1% BHT in tightly closed tubes in the dark. Thereafter, the mixture was separated using a centrifuge at 3,420 g for 15 min at 25°C. After separation, the residue was rinsed with three additional 5 mL volumes of the extraction solvent and centrifugation was done as above. Thereafter, the supernatants were pooled, dried with anhydrous sodium sulphate, filtered with a Whatman philtre paper (No 1), and evaporated to dryness under a stream of nitrogen. The extracts were re-dissolved in 1 ml of isopropyl alcohol (10%) in n-hexane and subsequently filtered through a 0.45 μm PTFE syringe philtre before analysis and determination. A Shimadzu Prominence-I LC-2030C 3D Liquid chromatograph equipped with an LC-2030 autosampler and LC-2020/2040 PDA detector (Shimadzu, Kyoto, Japan) was used. The column was a 250 mm × 4.6 mm id, 5 μm Shim pack GIST NH_2_ column. The wavelength was set at 460 nm at 30°C and the injection volume was 10 μL at a flow rate of 0.6 ml min^−1^. Identification of β-carotene was based on a comparison of retention times and absorption spectra with that of a pure standard. Quantification was done using a pure standard of β-carotene (LOD 13.82 and LOQ 46.06) and the standard curve ranged from 0 to 100 μg mL^−1.^

### Volatile Compounds

Volatile constituents were extracted according to the method of Hijaz et al. (18) with a few modifications. A leaf sample (2 g) was added to a 50 mm tube and 50 μl of internal standard butylated hydroxytoluene (1 mg ml^−1^), saturated sodium chloride (2 ml) were pipetted into the sample. Volatile compounds were extracted using n-hexane (2 ml) and the mixture was sonicated (MRC Ultrasonic cleaner, Model DC-150H, Health care technologies, Cape Town, South Africa) for 30 min at 30°C, thereafter, centrifuged (Hermle Labortechnik, Germany Type 2326K, 2010) at 3,000 *g* for 10 min and the supernatant was separated. The residue was rinsed three times with 2 mL of hexane and centrifuged as above. The supernatants were pooled and dried with sodium sulphate, filtered with a Whatman philtre paper (No1) and concentrated to 250 μL under a gentle stream of nitrogen, and stored at 5°C before analysis using an Agilent 7890A gas chromatograph (Agilent, Santa Clara, USA) hyphenated to an Agilent 5975 C MSD with a Triple axis detector to analyse the volatile compounds present in coriander leaves. The machine was equipped with an autosampler (Agilent Technologies GC Sampler 80). Helium was used as the carrier gas at a constant flow rate of 1 mL min^−1^. Separation was done on a Zebron Guardian Capillary GC column (USA) with dimensions 30 mm × 0.25 mm i.d and 0.25 μm film thickness. The chromatographic conditions were as follows: oven programme, 70°C for 1 min then 3°C min^−1^ to 142°C for 0 min then 5°C min^−1^ to 225°C for 3 min, then, 25°C min^−1^ to 320°C for 3 min, the total run time was 51.4 min. The splitless injection was carried out at a pressure of 61.6 kpa and an inlet temperature of 280°C. The mass detector was operated with an electron energy of 70 eV in electron ionisation mode. The ion source and quadrupole temperatures were 230 and 150°C, respectively. The injection volume was 1 μL. Compounds were identified using the mass spectral library (NIST mass spectral library, Version 8). Aldehydes were quantified using octanal reference standard [LOD (limits of detection) 3.73 and LOQ (limits of quantification) 12.45] plotted at concentrations ranging from 0 to 100 μg mL^−1^.

### Statistical Analysis

The samples were laid out in a completely randomised block design. The data were analysed using two-way ANOVA, and the means were separated using Fisher's protected LSD test (Least significant difference). The experiment was repeated twice to confirm the obtained data.

## Results and Discussion

### Effects of LED Lights on Mass Loss

**Figure 2:**
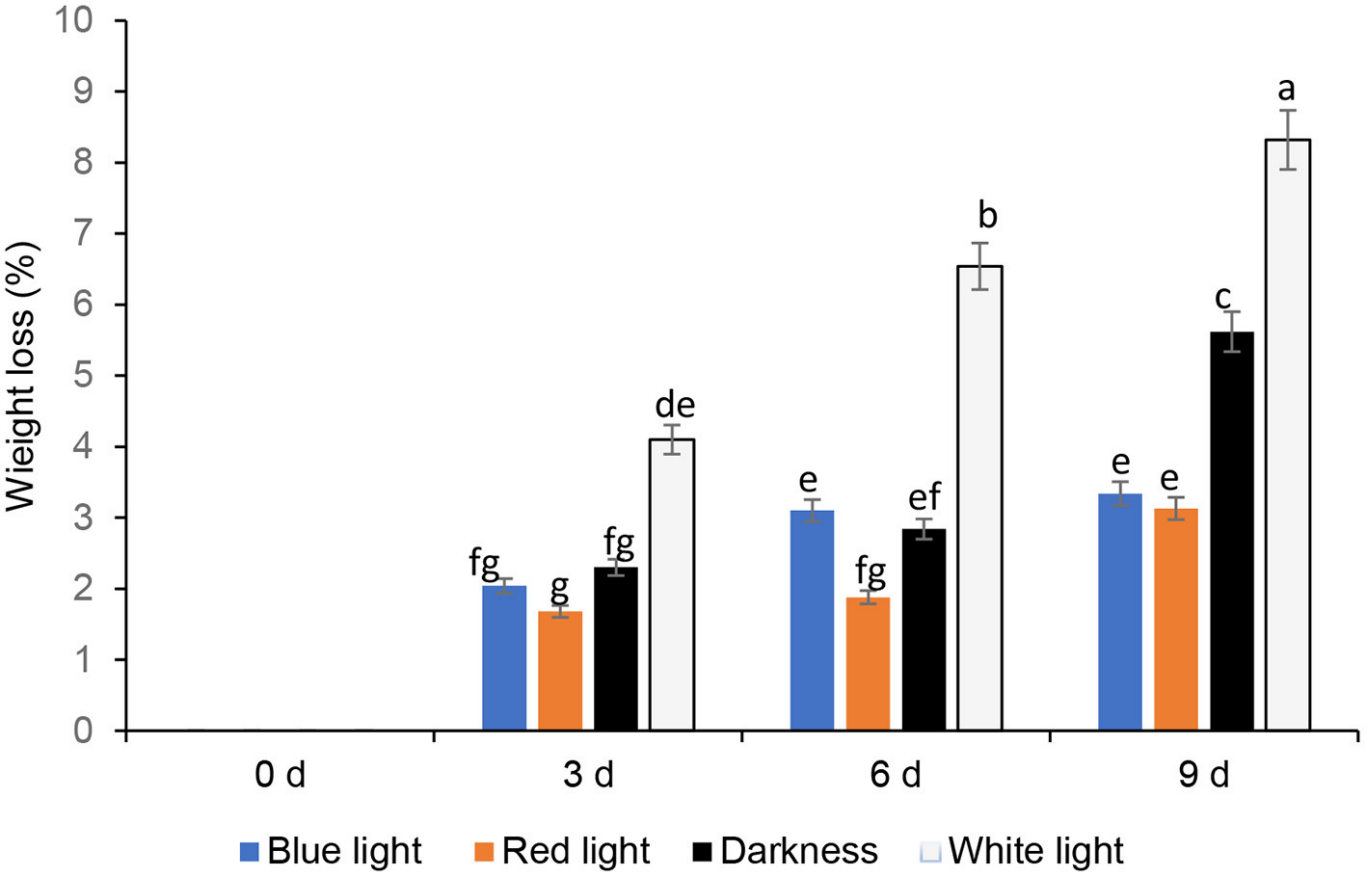
Effect of blue and red LED light treatment on mass loss of coriander (*Coriandrum sativum*) leaves after post-harvest storage. Bars with the same alphabetic letter along the column are not significantly different at *p* ≤ 0.05.

The mass loss of fresh coriander leaves exposed to different LED lights for 2 h and stored under cold storage for 3, 6, and 9 days is expressed in percentages (Figure 2), with darkness and white lights for 24 h, as controls. Leafy vegetables become unmarketable if they encounter a moisture loss of more than 5% of the original fresh mass (19). The results revealed that the coriander leaves exposed to red and blue LED lights for 2 h showed commercially allowable mass loss of up to 9 days compared to the other treatments. Coriander leaves exposed to white light and stored for 6 and 9 days showed significantly higher mass loss (>5%), which is commercially not acceptable. Favre et al. (20) demonstrated that the duration of the pulse of low-intensity white light increased the mass loss of broccoli heads during post-harvest storage. Furthermore, mass loss (%) of fresh vegetables is due to evaporation of water, dehydration, and respiration, which contributes to the breakdown of carbohydrates to produce carbon dioxide and water (21). Generally, an increase in light intensity increases the stomatal index (22). Kinoshita et al. (23) showed that blue light triggers stomatal opening leading to higher respiration rates and increased mass loss. This could be the reason for the observed higher mass loss on day 6 under the blue LED light compared to the red LED light (24). Jiang et al. (25) also showed that the red LED irradiation reduced the mass loss in broccoli. On day 9, however, there was no significant difference in mass loss between leaves stored under red and blue LED lights. The significant mass loss in white lights corroborates Braidot et al. (26) reports, as continuous or prolonged lights initiated adverse effects on white and green vegetables stored at low temperatures. High rates of respiration, compensated by significant photosynthetic activity, encourage increased mass loss under the white lights. Lee et al. (27) reported no significant differences between darkness and LED treatments after 18 days in terms of mass loss, and in this study, darkness reduced mass loss compared to white lights. Conversely, Zhan et al. (28) reported that light exposure accelerates mass loss compared to low-temperature storage in darkness in vegetables.

### Effects of LED Lights on Colour Change (ΔE)

The L^*^ (lightness) increased significantly with storage time with all light treatments included in this study. An increase of L^*^ indicates the leaf colour becoming more towards yellow and less retention of green colour. Likewise, the colour value a^*^ of the leaves subjected to different light treatments decreased with storage time. colour coordinate a^*^ is associated with the green colour (Kasajima) of the leaves and the degradation of the original green colour is evident from the colour value a^*^. The Colour coordinate b^*^ value represents the blue–yellow opponents, with negative numbers towards blue and positive towards yellow (29). The b^*^ colour value also increased with storage time, irrespective of the light treatments but the highest b^*^ value was noted in the leaves stored for 9 days under the white light indicating higher leaf yellowing. The colour change (Δ*E*) was reduced in coriander leaves exposed to blue LED light for 2 h and stored for 3–9 days compared to those that were exposed to the red LED light (2 h) and the controls (Table 1). Although the Δ*E* value increased in coriander leaves exposed to blue LED light with increasing storage time, leaves stored for 3 days showed the lowest Δ*E* value (Table 1).

**Table 1:** Influence of blue and red light-emitting diode (LED) lights for 2 h during storage on colour changes of coriander (*Coriandrum sativum*) leaves.

**Light type X light duration**	**Storage period**	L*	a*	b*	***ΔE***
Freshly harvested	0	35.79 ± 0.15g	−9.49 ± 0.16h	11.15 ± 0.25j	
Blue light	3	37.40 ± 0.11f	−10.44 ± 0.37g	13.24 ± 0.01h	1.30 ± 0.33l
Blue light	6	39.25 ± 0.23 e	−10.27 ± 0.24g	13.52 ± 0.41h	2.66 ± 0.26k
Blue light	9	41.07 ± 0.42cd	−12.30 ± 0.09c	15.91 ± 0.64de	5.42 ± 0.15g
Red light	3	38.17 ± 0.30 f	−10.17 ± 0.41g	12.81 ± 0.59i	3.15 ± 0.95j
Red light	6	40.08 ± 0.06de	−10.94 ± 0.30f	14.70 ± 0.77g	5.50 ± 0.34g
Red light	9	40.91 ± 0.12 d	−11.89 ± 0.36d	16.63 ± 0.14bc	6.73 ± 0.36f
Darkness	3	37.52 ± 0.12 f	−11.88 ± 0.28d	13.71 ± 0.40h	4.02 ± 0.54i
Darkness	6	41.47 ± 0.21 cd	−12.03 ± 0.27cd	15.16 ± 0.56f	7.52 ± 0.15e
Darkness	9	42.81 ± 0.12b	−12.66 ± 0.08b	15.95 ± 0.06de	9.09 ± 0.15d
White light	3	39.70 ± 0.09e	−10.61 ± 0.60e	13.42 ± 0.93h	4.86 ± 1.12h
White light	6	41.03 ± 0.35cd	−11.41 ± 0.63e	15.72 ± 1.12e	7.34 ± 1.35e
White light	9	44.84 ± 0.09a	−12.11 ± 0.26cd	16.94 ± 0.48b	10.27 ± 0.70c

*Values with the same alphabetic letter along the column are not significantly different at p ≤ 0.05*.

### Effects of LED Lights on Total Chlorophyll Content

**Figure 3:**
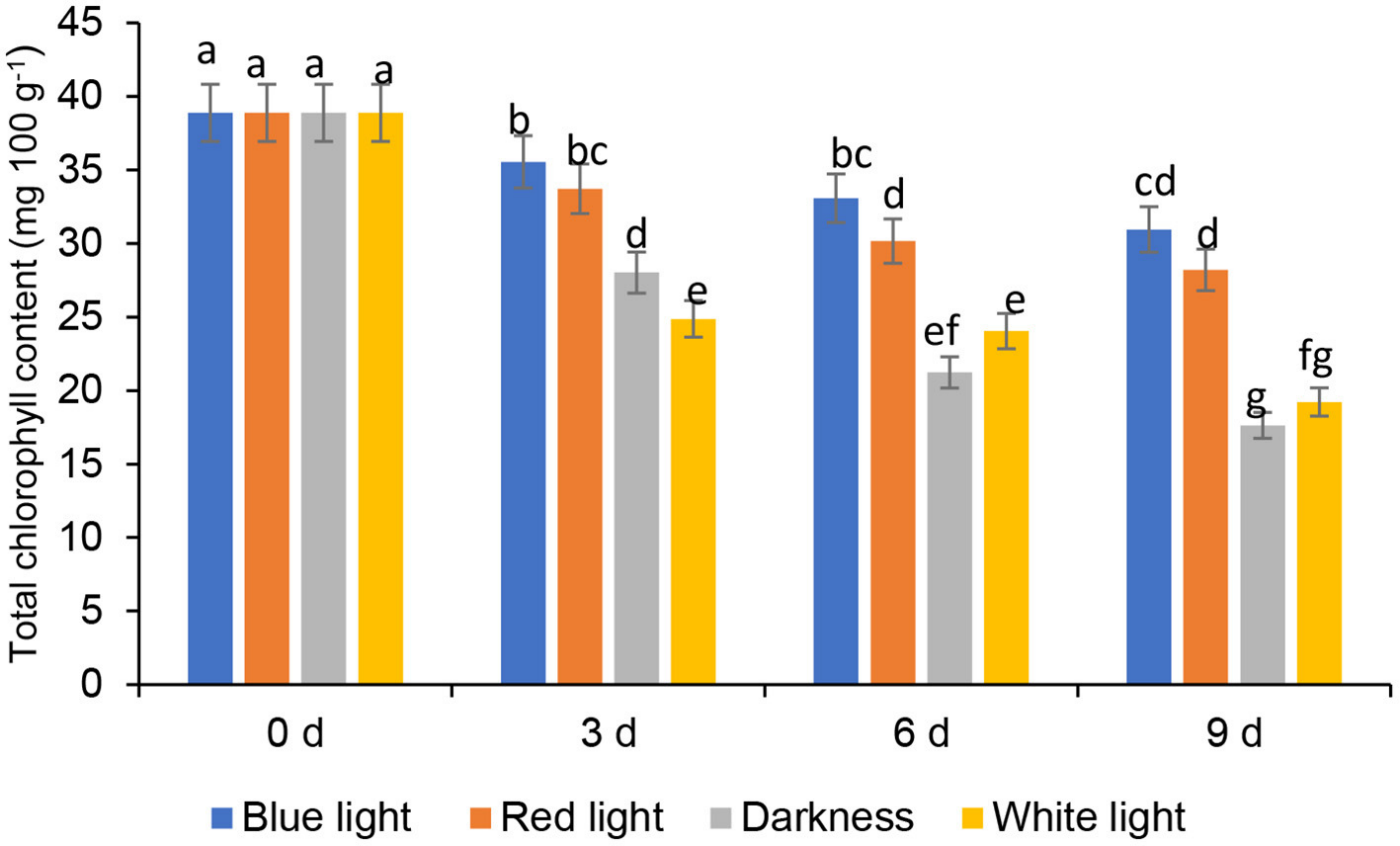
Effect of blue and red LED light treatment on total chlorophyll content in coriander (*Coriandrum sativum*) leaves after post-harvest storage. Bars with the same alphabetic letter along the column are not significantly different at *p* ≤ 0.05.

Chlorophyll levels in controls and the leaves exposed to red or blue LED light decreased significantly throughout the storage (Figure 3). Coriander leaves exposed to blue LED light reduced the total chlorophyll content by 8.56, 14.94, and 20.39% on days 3, 6, and 9, respectively, compared to the samples at harvest (0-day storage). On the other hand, the leaves exposed to the red LED light showed the loss of chlorophyll content by 13.27, 22.42, 27.46% on days 3, 6, and 9, respectively, compared to those at 0-day storage. However, the leaves from the controls showed a significant loss of chlorophyll content on days 3, 6, and 9 compared to the blue or red LED light treatments (Figure 3). Therefore, the blue and red light exposure had remarkably reduced the loss of total chlorophyll compared to the leaves from control treatments. Song et al. (30) showed that, in storage, blue LED light slightly inhibited the chlorophyll degradation in pak choi. Additionally, Li et al. (31) also showed that blue light (430 nm and 465 nm) remarkably influenced the improvement of chlorophylls in Chinese kale and pak choi baby leaves. According to Li et al. (31), degradation of chloroplast proteins is associated with senescence and a reduction in soluble protein content, and pre-harvest supplemental blue light treatment improved the accumulation of higher soluble protein content than control in the flower stalk of Chinese kale during storage. On the other hand, Jiang et al. (25) showed that the red LED irradiation retained the sensory attributes of broccoli, inhibited yellowing and the degradation of chlorophyll. In harvested coriander leaves, yellowing of leaves are an indication of quality degradation due to chlorophyll degradation. Chlorophyllase and Pheophorbide an oxygenase (PAO) are responsible for degrading chlorophyll in vegetables (32). Furthermore, LED lights were shown to reduce cell membrane damage and strengthen the membrane integrity in vegetables and also stimulate the antioxidant defence systems in plants inhibiting the accumulation of reactive oxygen species (ROS) and inhibiting oxidative damage. In addition, Jiang et al. (25) stated in their finding on the gene expression of LED light on the gene expression of chlorophyllase and PAO in broccoli, which was effective until the 3rd day. However, further investigations are needed to elucidate the underpinning of the mechanisms related to the positive impact of blue and red LED light on the reduction of chlorophyll content in coriander leaves.

### Effects of LED Lights on β Carotein Content

**Figure 4:**
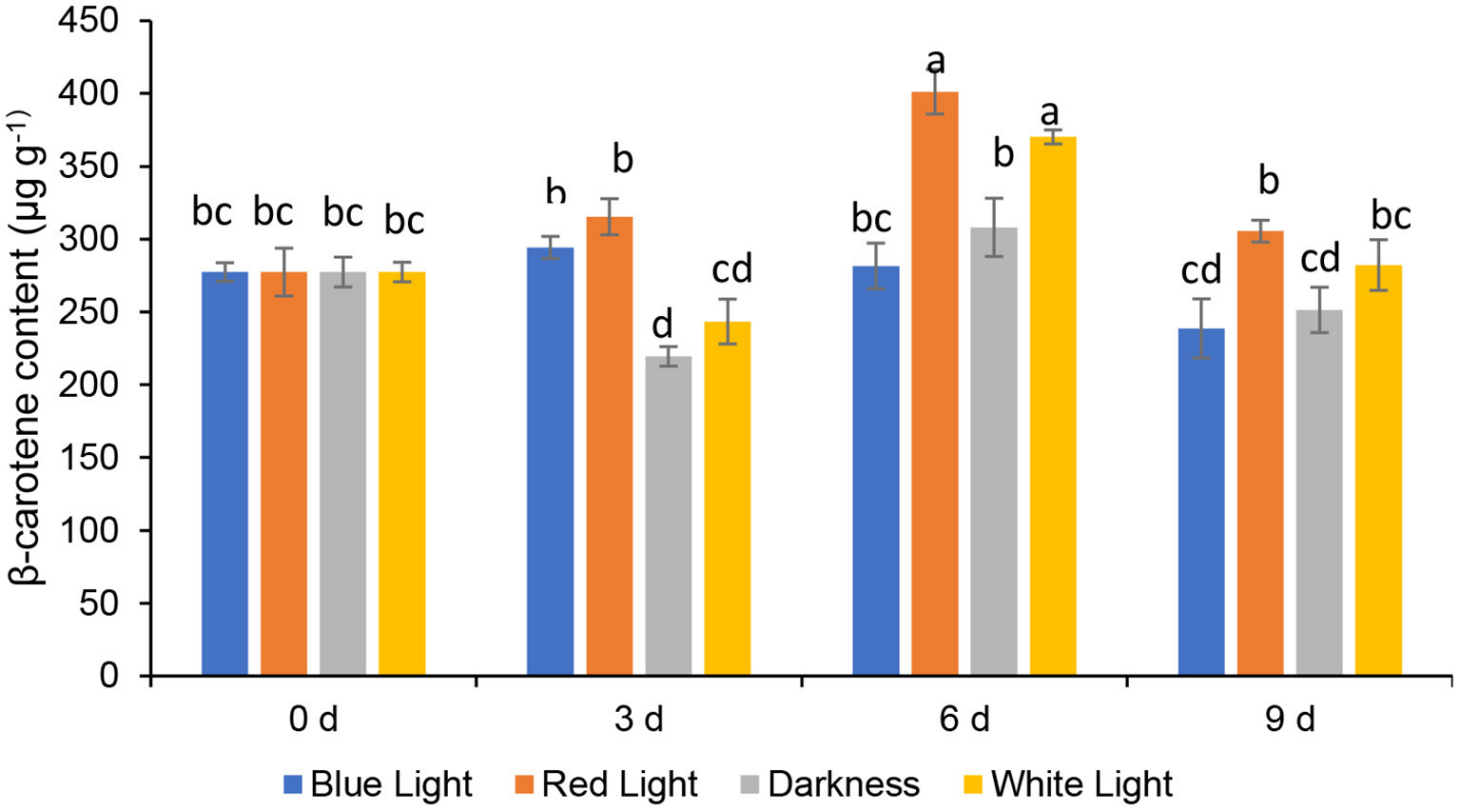
Effect of blue and red LED light treatment on β- carotene content coriander (*Coriandrum sativum*) leaves. Different letters in the same bar indicate significant differences at *p* < 0.05.

The β-Carotene, a provitamin A carotenoid in fruit and leafy vegetables, is vital due to its antioxidant properties to add value to consumers' diets (33). The β-carotene contents of coriander leaves, control samples (stored in the continuous darkness and white light for 2 h) and treated with LED red or blue lights for 6 h during storage up to 3, 6, and 9 days are presented in Figure 4. The β*-*carotene contents of coriander leaves exposed to the red LED light for 2 h, slightly increased (13.69%) on day 3, and thereafter significantly (*p* < 0.05) peaked by 44.55% on day 6 and declined on day 9. However, the β-carotene levels in leaves exposed to the red LED light for 2 h on days 3 and 9 did not differ significantly. Similarly, the coriander leaves exposed to white light (2 h) showed a significant increase of 33.45% on day 6 and, subsequently, declined on day 9, remaining similar to the levels of day 0 samples. On the other hand, coriander leaves stored in continuous darkness showed similar levels of β-carotene content as freshly harvested leaves (day 0) on day 6. β-carotene content in coriander leaves stored under blue LED lights declined significantly on day 9 by 13.95%. LED red light showed an increase in the β-carotene in parsley, while it decreased in basil (34). Therefore, the species-dependent carotenoid response was evident and could be related to the conversion of one carotenoid to another carotenoid, which had played a major role in the accumulation of β-carotene (35) on day 6 under LED red light and normal white light. However, further research is needed in terms of gene expression analysis of βLCY: β-cyclase (35), responsible for the accumulation of β-carotene.

### Effects of LED Lights on Total Phenols and Phenolic Components

**Figure 5:**
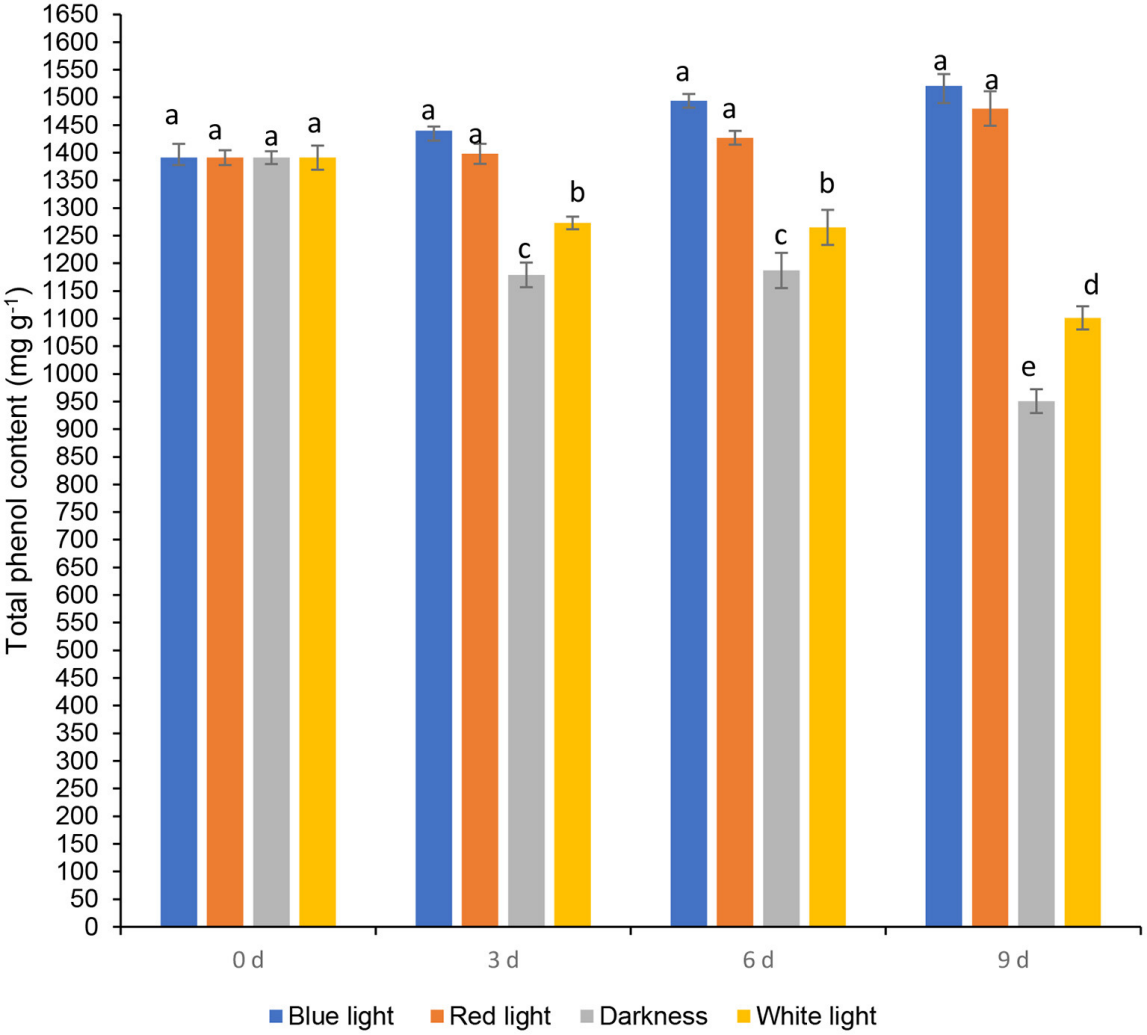
Effect of blue and red LED light treatment on β-carotene content coriander (*Coriandrum sativum*) leaves. Different letters in the same bar indicate significant differences at *p* < 0.05.

Total phenol content in coriander leaves exposed to the blue LED light insignificantly increased on day 6, by 7.39% compared to the samples at harvest (day 0) (Figure 5). Whilst an insiginificant increase in total phenolic content was noted in leaves exposed to blue and red LED light by 9.34 and 6.39% on day 9, respectively. Coriander leaves stored in darkness or exposed to white light for 2 h during storage for 6 and 9 days resulted in a significant loss of total phenolic content. Red LED light caused an increase of phenolics in baby leaf lettuce (36) and blue LED light improved the concentration of phenolic compounds in tomatoes (37). Routray et al. (38) showed an increase in phenolic content of the leaves treated with blue LED light during post-harvest storage. Moreover, red and blue LED lights improved the total phenolic compounds in yellow sweet peppers (7 days), green sweet peppers, and red sweet pepper (14 days) respectively during post-harvest storage at 7°C (10). Maroga et al. (10) showed the activation of phenylalanine ammonia-lyase enzyme (PAL), an activity that is responsible for the biosynthesis of secondary metabolites (phenols and flavonoids) *via* the phenylpropanoid pathway.

Folin-Ciocalteu assay is the method most frequently used to determine total phenolic content in food products, since it is a rapid and convenient method (39). Despite this, the Folin-Ciocalteu assay is not specific to total phenolic content determinations. Ascorbic acid and reducing sugars are also found in plant extracts and are responsive to the Folin-Ciocalteu assay (39). Therefore, the investigation was conducted to see the influence of LED lights on different phenolic components using HPLC-DAD in this study.

**Figure 6:**
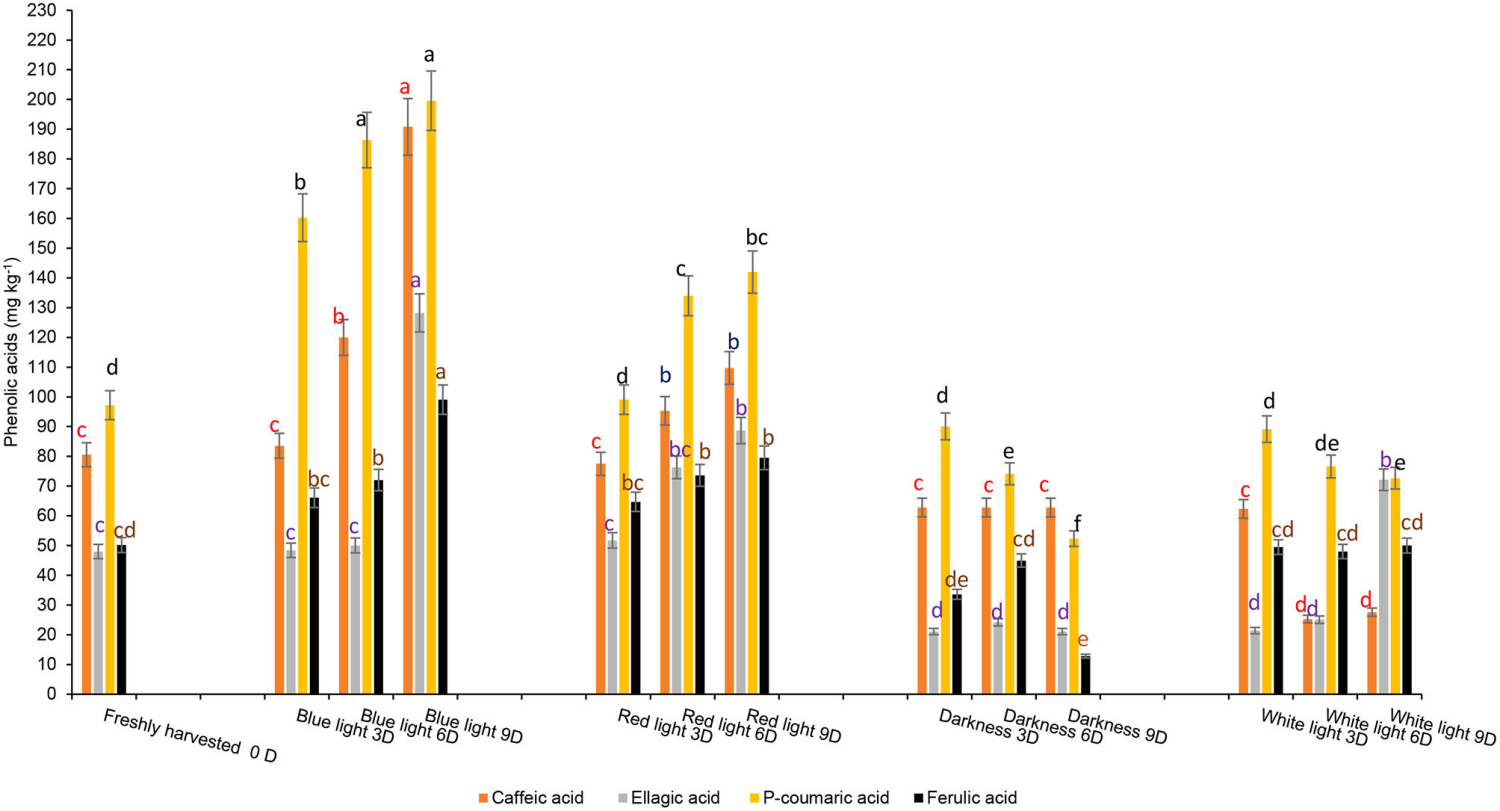
Effect of blue and red LED light treatments on the changes in the concentration of major phenolic acids in coriander (*Coriandrum sativum*) leaves. Different letters in the same bar indicate significant differences at *p* < 0.05 for a specific phenolic acid.

**Figure 7:**
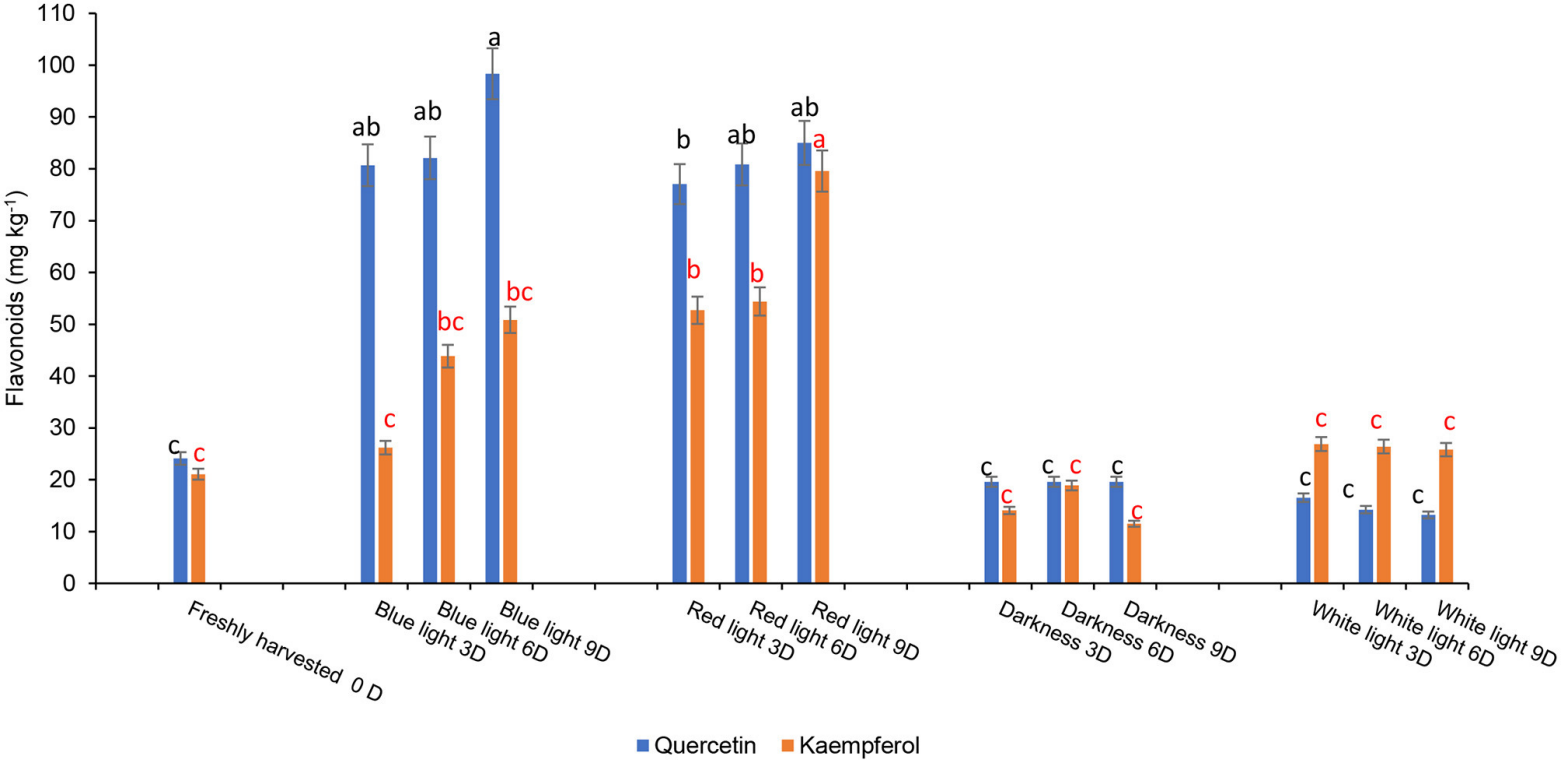
Effect of blue and red LED light treatments on the changes in the concentration of major flavonoids in coriander (*Coriandrum sativum*) leaves. Different letters in the same bar indicate significant differences at *p* < 0.05.

The influence of red or blue LED light treatments and controls on different phenolic components are shown in Figure 6. Fresh coriander leaves contained caffeic acid (80.55 mg kg^−1^), ellagic acid (48 mg kg^−1^), p-coumaric acid (97.2 mg kg^−1^), ferulic acid (50.16 mg kg^−1^), quercetin (24.1 mg kg^−1^), and kaempferol (21.07 mg kg^−1^). Coriander leaves exposed to blue and red LED light for 2 h over 6 and 9 days in cold storage showed significant increases in caffeic acid, ellagic acid, p-coumaric acid, and ferulic acid content when compared to the control treatments and freshly harvested leaves. In contrast, the levels of caffeic acid (190.79 mg kg^−1^), ellagic acid (128.14 mg kg^−1^), p-coumaric acid (199.6 mg kg-1), and ferulic acid (99.06 mg kg^−1^) were highest on day 9 of cold storage under the exposure of blue LED light for 2 h compared to controls and freshly harvested leaves. Likewise, the levels of flavonoids, quercetin, and kaempferol were significantly higher in the coriander leaves exposed to blue or red LED light for 3, 6, or 9 days in cold storage in comparison to the controls and freshly harvested leaves (Figure 7). However, on day 9 of cold storage, leaves exposed to blue LED light reached quercetin content (98.35 mg kg^−1^), while those exposed to the red LED light had the highest concentration of kaempferol (79.57 mg kg^−1^). A similar observation was reported in the onion bulb and pulp held under the blue light showing the highest quercetin glucosides, whereas the onions stored under the white fluorescent light showed the lowest content (40). For the biosynthesis of phenolic compounds, the carbohydrates are converted into phenylalanine, tyrosine, and tryptophan (intermediate amino acids) *via* the shikimate pathway (41). For phenolics biosynthesis, the process starts by changing carbohydrates into intermediate amino acids (i.e., phenylalanine, tyrosine, and tryptophan) through the shikimate pathway by the action of 3-deoxy-D-arabinose-heptulosonate 7-phosphate or DAHP synthase, chorismate mutase, and anthranilate synthase (41). The LED blue light treatments could have induced the activation of the shikimate pathway to increase the concentration of the precursors for the production of different phenolic compounds *via* the phenylpropanoid pathway (41). Red LED light increased the biosynthesis of flavonoid (epicatechin) in avocados by inducing the upregulation of phenylalanine ammonia-lyase gene expression (11). However, Liu et al. (42) showed that compared to red light, the blue light-induced a higher flavonoid accumulation in *Cyclocarya paliurus* leaves, especially key health-promoting flavonoids such as kaempferol, and quercetin.

### Effects of LED Lights on Antioxidant Properties

Antioxidant properties of different vegetables including Chinese cabbage, kale (43), and tomato (44) were improved when exposed to blue LED light and in avocado by the red LED light (9). DPPH•+ radical-scavenging activity increased significantly in coriander leaves exposed to 6 h of blue LED light during storage for 9 days (Table 2). The highest antioxidant activity against the ABTS radical was obtained in leaves exposed to blue LED and red LED light for 6 or 9 days and 3–9 days, respectively (Table 2). Conversely, the highest FRAP activity was detected in leaves exposed to blue LED light for 9 days (Table 2). A correlation analysis carried out with the results obtained using FRAP, ABTA•+, and DPPH•+ antioxidant assays showed higher correlations between *p*-coumaric acid (*r* = 0.82, *p* < 0.05), caffeic acid (*r* = 0.75, *p* < 0.05), and quercitin (*r* = 0.70, *p* < 0.05) showed a strong positive correlation with FRAP activity. The *p*-coumaric acid generally gave more ABTS•+ and DPPH•+ radical scavenging potency; this is because of resonance stabilisation in the hydroxycinnamic acids enhanced by the conjugation between π electrons of the ring and the π bond of the side chain (45). The presence of the electron-donating hydroxyl group attached to the benzene ring in *p*-coumaric acid improved its ABTS•+, DPPH•+ radical scavenging, and its antioxidant activity as shown by the good FRAP correlation. Ferulic acid has the hydroxyl group and another electron-donating methoxy group attached to the benzene ring which makes it a better ABTS and DPPH radical scavenger and better antioxidant as shown in the FRAP assay (strong correlation) than *p* coumaric acid.

**Table 2:** The influence of red and blue LED lights on the antioxidant properties of coriander leaves during post-harvest storage for 3–9 days.

**Light type X light duration**	**Storage period**	**DPPH (IC_50_ μg mL^−1^)**	**ABTS (IC_50_ μg mL^−1^)**	**FRAP (mM TEAC g^−1^)**
Freshly harvested	0	534.53 ± 1.41f	280.44 ± 4.76ab	13.7 ± 0.75d
Blue	3	477.94 ± 1.54g	250.47 ± 2.59ab	8.8 ± 1.7f
Blue	6	446.31 ± 1.44gh	201.37 ± 3.06c	16.7 ± 1.6b
Blue	9	392.89 ± 1.45h	183.67 ± 4.44c	19.0 ± 0.2a
Red	3	498.44 ± 1.70fg	247.65 ± 1.88bc	14.8 ± 1.1cd
Red	6	485.18 ± 1.36fg	231.33 ± 0.58bc	15.6 ± 0.2bc
Red	9	455.60 ± 8.74g	193.22 ± 0.70c	15.9 ± 0.4bc
Darkness	3	628.32 ± 1.74e	293.27 ± 1.79ab	11.9 ± 1.7e
Darkness	6	767.45 ± 1.19d	297.65 ±1.23ab	8.4 ± 1.5f
Darkness	9	1,087.47 ± 1.81a	316.24 ± 3.90a	5.0 ± 0.4h
White light	3	875.64 ± 1.46c	231.81 ± 3.45bc	12.4 ± 0.3e
White light	6	952.73 ± 1.78b	231.90 ± 3.16bc	11.7 ± 0.8e
White light	9	900.06 ± 1.99bc	255.83 ± 3.92ab	6.9 ± 2.5g

*Values with dissimilar letters in a column are significantly different at p ≤ 0.05. Coriander leaves were exposed to 6 h of red or blue LED light, Control -continuous darkness, and those leaves were exposed in 2 h white light*.

### Effects of LED Lights on Volatile Compounds and Overall Acceptance

**Figure 8:**
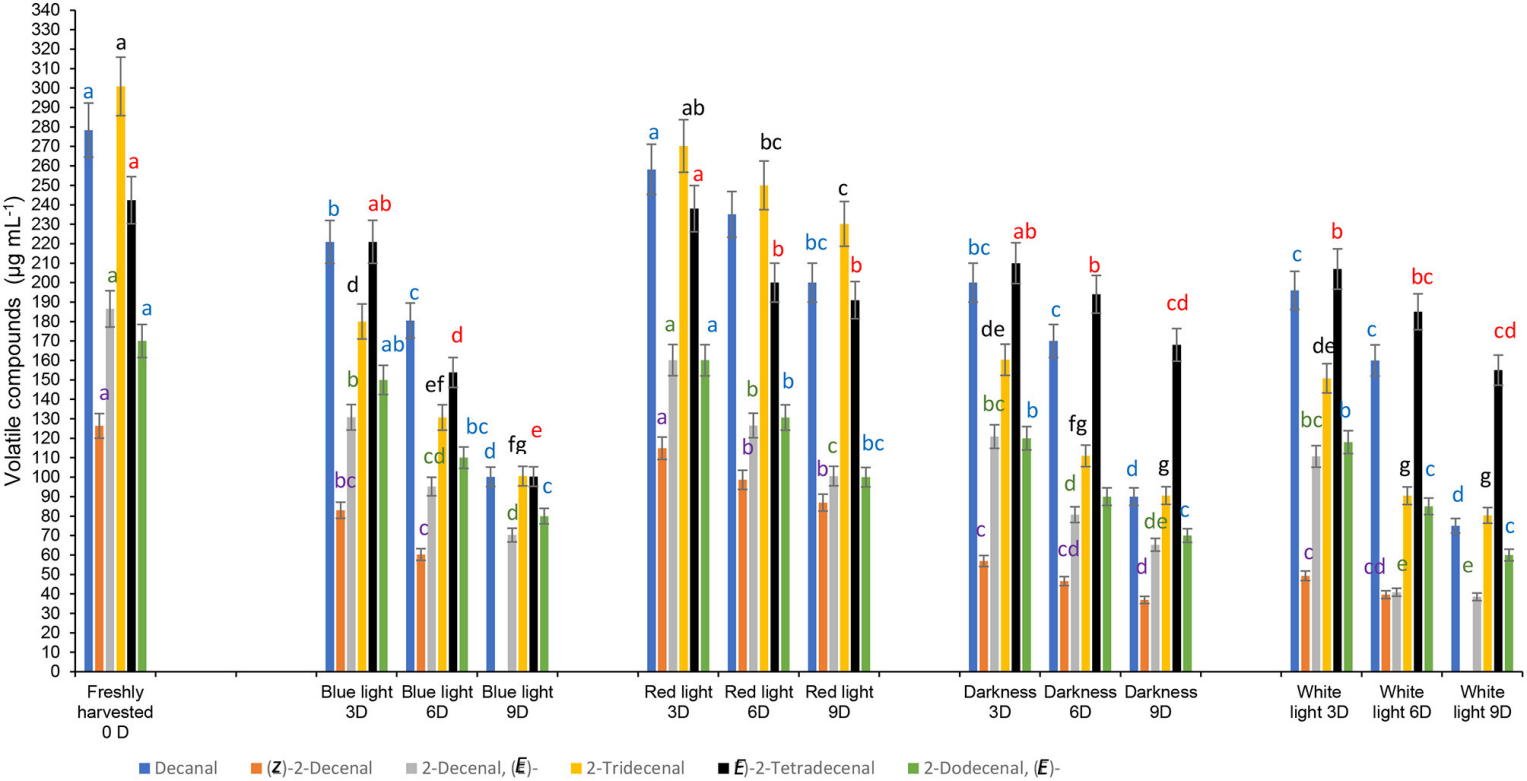
Effect of blue and red LED light treatments on the changes in the concentrations of major volatile compounds in coriander (*Coriandrum sativum*) leaves. Different letters in the same bar indicate significant differences at *p* < 0.05 for a specific volatile compound.

The aroma of coriander leaves is important to determine their quality. Aliphatic aldehydes, decanal (114.2 μg mL^−1^), 2-decenal, (*E*)- (140.78 μg mL^−1^), and (*E*)-2-tetradecenal (54.01 μg mL^−1^) were also detected in moderately higher concentrations. Figure 8 shows the results on the composition of predominantly detected aroma volatile compounds exposed to different LED light treatments after storage at 3, 6, and 9 days compared to the controls. Coriander leaves exposed to red light for 2 h during 3 days of cold storage retained the concentrations of aroma volatiles, decanal, 2-decenal, (*E*)-, (Z)-2-decenal, 2-tridecenal (*E*)-2-tetradecenal and 2-dodecenal, (*E*) similar to the freshly harvested leaves. In general, the levels of aroma volatile compounds decreased with storage time. In contrast, the leaves exposed to the red LED light for 2 h and stored for 6 and 9 days in cold storage showed higher levels of all six aroma compounds than the samples exposed to blue LED light or white light or stored in darkness.

However, aroma compounds may differ in their odour perception thresholds, thus, the higher concentrations do not always influence the olfactory impact. Previously, McAusland et al. (46) showed that the red LED light improved the increase of (*E*)-2-tridecenal and dodecanal significantly during growing coriander under a controlled environment chamber, which differed from our findings.

Furthermore, McAusland et al. (46) stated that the odour notes of 2-dodecanal and (*E*)-2-tridecenal are waxy, aldehydic, citrus, and orange rind with floral nuances” and “sweet” scents. McAusland et al. (46) showed that these aldehydes have a smaller odour threshold, thus, at greater concentrations, these compounds contribute more to the odour or scent of the fresh coriander leaves. A large portion of plant volatile compounds was derived from saturated and unsaturated fatty acids. Straight chain aldehydes are formed by three processes, α-oxidation, β-oxidation, and the action of lipoxygenase. Buthelezi et al. (13) had shown the interference of spectral quality at the pre-harvest stage on the volatile compounds after post-harvest storage and argued possibly the conversion of the inactive phytochrome Pr to the biologically active Pfr had influenced the lipoxygenase activity. In this study, although exposure to the red LED light for 2 h during cold storage delayed the loss of aroma compounds compared with blue LED light and the control treatments, the interference of red LED light on volatile compounds was minimal.

**Figure 9:**
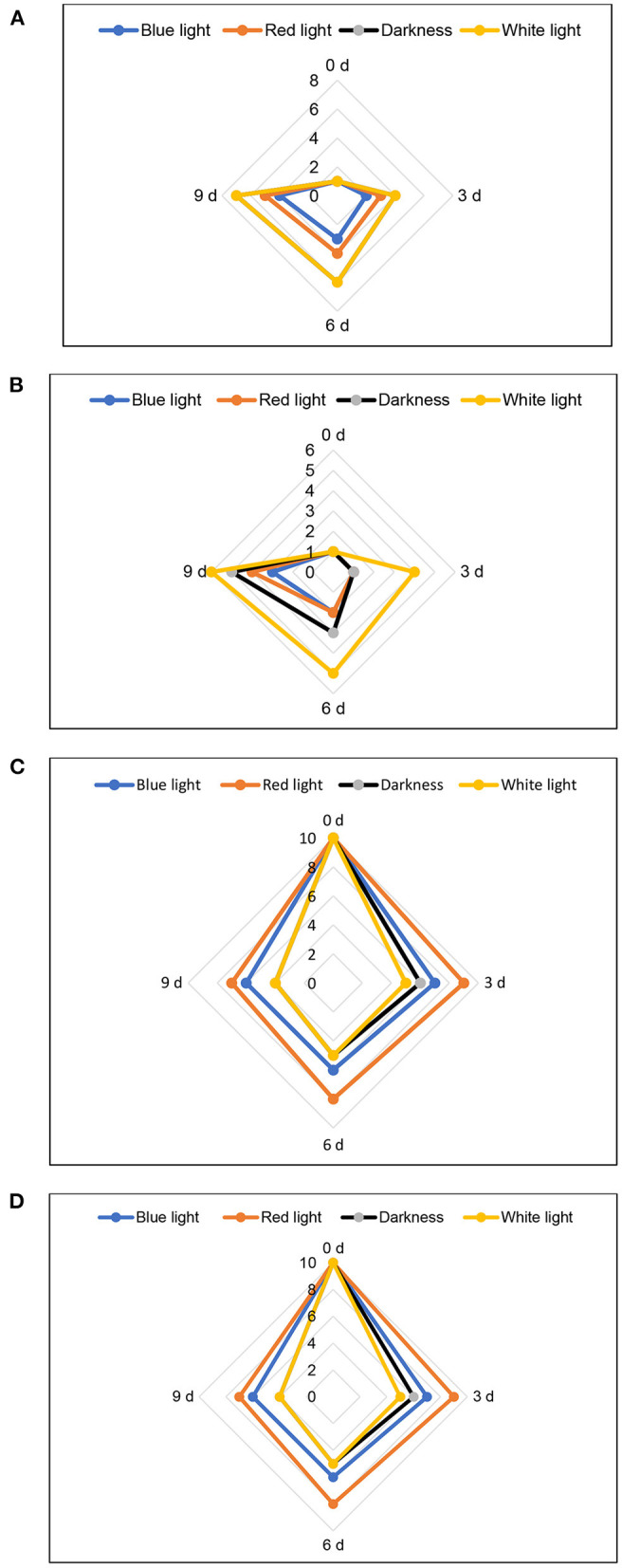
Influence of blue and red LED light treatment on **(A)** yellowing, **(B)** shrivelling, **(C)** typical aroma, and **(D)** overall acceptability of coriander leaves. For shrivelling and yellowing; 10–9 = very bad 50% unmarketable, 8–7 = poor 25% yellowing/shrivelling, unmarketable; 6–5 = average 10% yellowing/shrivelling, limited marketability; 4–3 = good, 1–5% yellowing/shrivelling, marketable; 1–2 = excellent. For typical aroma and overall acceptance; 10–9 = excellent, 8–7 = good, marketable; 6–5 = average, limited marketability; and 4–3 = poor, unmarketable.

Sensory panellists detected slight yellowing of leaves from control samples and those exposed to 2 h red LED light treatment after cold storage on day 9 (score 6; 10% yellowing), while samples exposed to blue LED light and stored in cold storage for 3–6 days scored 1–2, indicating the absence of yellowing (Figure 9A). Panellists rated the leaves exposed to blue LED light as “good” (score of 4) on day 9 of cold storage. In contrast, there was marked shrivelling in coriander leaves exposed to white light for 2 h on day 3 onwards, and less shrivelling was observed (score 3–4) in leaves exposed to red and blue LED light on day 9 of cold storage (Figure 9B). The panellist score for typical aroma remarkably declined on days 6 and 9 and all treatments but the red light showed a score of 8–7 (still “good”). On day 9, samples from control treatments severely affected the aroma (score 4, poor). Finally, the overall acceptance score by the panellists declined with storage time. On day 3, the overall acceptance score was 8 and 7 for coriander leaves exposed to blue and red LED lights, respectively, indicating that the samples from all four treatments were “good” (Figure 9C). On day 6, the samples from the blue LED light treatment showed scores of 8 and 6 indicating that the samples were still in “good” condition. Based on the panelists' data, on day 9, the sample from the blue LED light treatment showed a score of 7 (good), while the samples exposed to the red LED light indicated limited marketability (score 5), and the control treatments, darkness, and white lights were unmarketable (Figure 9D). It is likely that the yellowing of the coriander leaves after days 6 and 9 of cold storage negatively impacted the overall acceptance of the leaves, due to increased yellowing and loss of chlorophyll content, as shown in Figure 4.

## Conclusion

This study demonstrated the practical application of using LED light as a post-harvest treatment to improve the antioxidant properties and retain the acceptable aroma profiles in coriander leaves to benefit the consumers. In addition, this study provided a method for extending the storage life up to 9 days by exposing the packed coriander leaves in PET punnets to blue LED lights for 2 h at 5°C during cold storage, which increased antioxidant properties and levels of typical coriander aroma compounds. The shelf life and health-promoting properties of coriander leaves can be enhanced by implementing this technology at the supermarket storage or retail shelves.

## Author Contributions

TS performed the analysis of volatile compounds and beta carotene. VM performed the antioxidant assays, statistical analysis, data validation, and visualization. MM conducted the physicochemical analysis. DS conceptualised the research, was the grant holder and project manager, and wrote the final draft of the article. All authors contributed to the article and approved the submitted version.

## Funding

Financial support was received from the NRF (National Research Foundation), grant number 98352, for the research programme on Phytochemical Food Network to Improve Nutritional Quality for Consumers.

## Conflict of Interest

The authors declare that the research was conducted in the absence of any commercial or financial relationships that could be construed as a potential conflict of interest.

## Publisher's Note

All claims expressed in this article are solely those of the authors and do not necessarily represent those of their affiliated organizations, or those of the publisher, the editors and the reviewers. Any product that may be evaluated in this article, or claim that may be made by its manufacturer, is not guaranteed or endorsed by the publisher.

## References

[1] Sahib NG Anwar F Gilani AH Hamid AA Saari N Alkharfy KM. Coriander (*Coriandrum sativum* L.): a potential source of high-value components for functional foods and nutraceuticals- a review. Phytother Res. (2013) 27:1439–56. 10.1002/ptr.4897 23281145

[2] El-Zaeddi H Calín-Sánchez A Nowicka P Martínez-Tomé J Noguera-Artiaga L Burló F. Preharvest treatments with malic, oxalic, and acetylsalicylic acids affect the phenolic composition and antioxidant capacity of coriander, dill and parsley. Food Chem. (2017) 226:179–86. 10.1016/j.foodchem.2017.01.067 28254010

[3] Shahwar MK El-Ghorab AH Anjum FM Butt MS Hussain S Nadeem M. Characterization of coriander (*Coriandrum sativum* L.) seeds and leaves: volatile and non volatile extracts. Int J Food Prop. (2012) 15:736–47. 10.1080/10942912.2010.500068

[4] Hassana FAS Mahfouz SA. Effect of 1-methylcyclopropene (1-MCP) on the post-harvest senescence of coriander leaves during storage and its relation to antioxidant enzyme activity. Sci Hortic. (2012) 141:69–75. 10.1016/j.scienta.2012.04.021

[5] O'Hare TJ Wong LS. Leafy Asian vegetables. Extending their shelf life: Part 2 Rural Industries Research Development Corporation Publication No. 02/006. (2002). Available online at: https://www.agrifutures.com.au (accessed March 23, 2020).

[6] Loi M Liuzzi VC Fanelli F De Leonardis S Creanza TM Ancona N. Effect of different light-emitting diode (LED) irradiation on the shelf life and phytonutrient content of broccoli (*Brassica oleracea* L. var italica). Food Chem. (2019) 283:206–14. 10.1016/j.foodchem.2019.01.021 30722863

[7] Xie C Tang J Xiao J Geng X Guo L. Purple light-emitting diode (LED) lights controls chlorophyll degradation and enhances nutraceutical quality of post-harvest broccoli florets. Sci Hortic. (2022) 294:110768. 10.1016/j.scienta.2021.110768

[8] Folta K Carvalho SD. Photoreceptors and control of horticultural plant traits. HortScience. (2015) 50:1274–80. 10.21273/HORTSCI.50.9.1274

[9] Bantis F Smirnakou S Ouzounis T Koukounaras A. Current status and recent achievements in the field of horticulture with the use of light-emitting diodes (LEDs). Sci Hortic. (2018) 235:437–51. 10.1016/j.scienta.2018.02.058

[10] Maroga GM Soundy P Sivakumar D. Different post-harvest responses of fresh-cut sweet peppers related to quality and antioxidant and phenylalanine ammonia lyase activities during exposure to light-emitting diode treatments. Foods. (2019) 9:359. 10.3390/foods8090359 31450777 PMC6769952

[11] Mpai S Sivakumar D. Stimulation of light-emitting diode treatment on defence system and changes in mesocarp metabolites of avocados cultivars (Hass and Fuerte) during simulated market shelf conditions. Agronomy. (2020) 10:1654. 10.3390/agronomy10111654

[12] Panjai L Röhlen-Schmittgen S Ellenberger J Noga GJ. Effect of post-harvest irradiation with red light on epidermal color and carotenoid concentration in different parts of tomatoes. J Food Meas Charact. (2021) 15:1–10. 10.1007/s11694-020-00770-0

[13] Buthelezi MND Soundy P Jifon J Sivakumar D. Spectral quality of photo-selective nets improves phytochemicals and aroma volatiles in coriander leaves (*Coriandrum sativum* L) after post-harvest storage. J Photochem Photobiol. (2016) 161:328–34. 10.1016/j.jphotobiol.2016.05.032 27295414

[14] Eyarkai Nambi V Gupta RK Kumar S Sharma PC. Degradation kinetics of bioactive components, antioxidant activity, colour and textural properties of selected vegetables during blanching. J Food Sci Technol. (2016) 53:3073–82. 10.1007/s13197-016-2280-2 27765978 PMC5052176

[15] Managa MG Remize F Garcia C Sivakumar D. Effect of moist cooking blanching on colour, phenolic metabolites and glucosinolate content in chinese cabbage (*Brassica rapa* L. subsp chinensis). Foods. (2019) 8:399. 10.3390/foods8090399 31500353 PMC6770643

[16] Seke F Manhivi VE Shoko T Slabbert RM Sultanbawa Y Sivakumar D. Effect of freeze drying and simulated gastrointestinal digestion on phenolic metabolites and antioxidant property of the Natal plum (*Carissa macrocarpa*). Foods. (2021) 10:1420. 10.3390/foods10061420 34207411 PMC8235007

[17] Panfili G Fratianni A Irano M. Improved normal-phase high-performance liquid chromatography procedure for the determination of carotenoids in cereals. J Agric Food Chem. (2004) 52:6373–7. 10.1021/jf0402025 15478994

[18] Hijaz F Nehela Y Killiny N. Possible role of plant volatiles in tolerance against huanglongbing in citrus. Plant Signal Behav. (2016) 11:e1138193. 10.1080/15592324.2016.1138193 26829496 PMC4883952

[19] Charles F Nilprapruck P Roux D Sallanon H. Visible light is a new tool to maintain fresh-cut lettuce post-harvest quality. Post-Harvest Biol Technol. (2017) 135:51–6. 10.1016/j.postharvbio.2017.08.024

[20] Favre N Bárcena A Bahima JV Martínez G Costa L. Pulses of low intensity light as promising technology to delay post-harvest senescence of broccoli. Post-harvest Biol Technol. (2018) 142:107–14. 10.1016/j.postharvbio.2017.11.006

[21] Moreira M Del R Ponce AG Del Valle CE Ansorena R Roura SI. Effects of abusive temperatures on the post-harvest quality of lettuce leaves: ascorbic acid loss and microbial growth. J Appl Hortic. (2006) 8:109–13. 10.37855/jah.2006.v08i02.25

[22] Casson S Gray JE. Influence of environmental factors on stomatal development. New Phytol. (2008) 178:9–23. 10.1111/j.1469-8137.2007.02351.x 18266617

[23] Kinoshita T Doi M Suetsugu N Kagawa T Wada M Shimazaki KI. Phot1 and phot2 mediate blue light regulation of stomatal opening. Nature. (2001) 414:656–60. 10.1038/414656a 11740564

[24] Hasperué JH Guardianelli L Rodoni LM Chaves AR Martínez GA. Continuous white blue LED light exposition delays post-harvest senescence of broccoli. LWT Food Sci Technol. (2016) 65:495–502. 10.1016/j.lwt.2015.08.041

[25] Jiang A Zuo J Zheng Q Guo L Gao L Zhao S. Red LED irradiation maintains the post-harvest quality of broccoli by elevating antioxidant enzyme activity and reducing the expression of senescence-related genes. Sci Hortic. (2019) 251:73–9. 10.1016/j.scienta.2019.03.016

[26] Braidot E Petrussa E Peresson C Patui S Bertolini A Tubaro F. Low intensity light cycles improve the quality of lamb's lettuce (*Valerianella olitoria* [L] Pollich) during storage at low temperature. Post-harvest Biol Technol. (2014) 90:15–23. 10.1016/j.postharvbio.2013.12.003

[27] Lee YJ Ha JY Oh JE Cho MS. The effect of LED irradiation on the quality of cabbage stored at a low temperature. Food Sci Biotechnol. (2014) 23:1087–93. 10.1007/s10068-014-0149-6

[28] Zhan L Hu J Li Y Pang L. Combination of light exposure and low temperature in preserving quality and extending shelf-life of fresh-cut broccoli (*Brassica oleracea* L). Post-harvest Biol Technol. (2012) 72:76–81. 10.1016/j.postharvbio.2012.05.001

[29] Kasajima I. Measuring plant colors. Plant Biotechnol. (2019) 36:63–75. 10.5511/plantbiotechnology.19.0322a 31768106 PMC6847779

[30] Song Y Qiu K Gao J Kuai B. Molecular and physiological analyses of the effects of red and blue LED light irradiation on post-harvest senescence of pak choi. Post-Harvest Biol Technol. (2020) 164:111155. 10.1016/j.postharvbio.2020.111155

[31] Li Y Zheng Y Zheng D Zhang Y Liu H. Effects of supplementary blue and UV-A LED lights on morphology and phytochemicals of Brassicaceae baby-leaves. Molecules. (2020) 25:5678. 10.3390/molecules25235678 33276420 PMC7729980

[32] Hörtensteiner S Kräutler B. Chlorophyll breakdown in higher plants. Biochim Biophys Acta Bioenerg. (2010) 1807:977–88. 10.1016/j.bbabio.2010.12.007 21167811

[33] Grune T Lietz G Palou A Ross AC Stahl W Tang G. β-Carotene is an important vitamin A source for humans. J Nutr. (2010) 140:2268–85. 10.3945/jn.109.119024 20980645 PMC3139236

[34] Samuoliene G Brazaityte A Virsile A Jankauskiene J Sakalauskiene S Duchovskis P. Red light-dose or wavelength-dependent photoresponse of antioxidants in herb microgreens. PLoS ONE. (2016) 11:e0163405. 10.1371/journal.pone.0163405 27677090 PMC5038936

[35] Frede K Schreiner M Baldermann S. Light quality-induced changes of carotenoid composition in pak choi *Brassica rapa* ssp Chinensis. J Photochem Photobiol B Biol. (2016) 193:18–30. 10.1016/j.jphotobiol.2019.02.001 30798151

[36] Li Q Kubota C. Effects of supplemental light quality on growth and phytochemicals of baby leaf lettuce. Environ Exp Bot. (2009) 67:59–64. 10.1016/j.envexpbot.2009.06.01123584932

[37] Kim K Kook HS Jang YJ Lee WH Kamala-Kannan S Chae JC. The effect of blue-light emitting diodes on antioxidant properties and resistance to *Botrytis cinerea* in tomato. J plant pathol microbiol. (2013) 4:203. 10.4172/2157-7471.1000203

[38] Routray W Orsat V Lefsrud M. Effect of post-harvest LED application on phenolic and antioxidant components of blueberry leaves. Chem Eng. (2018) 2:56. 10.3390/chemengineering2040056

[39] Muñoz-Bernal ÓA Torres-Aguirre GA Núñez-Gastélum JA de la Rosa LA Rodrigo-García J Ayala-Zavala JF. New approach to the interaction between Folin-Ciocalteu reactive and sugars during the quantification of total phenols. Rev Espec Cienc Quim-B. (2017) 20:23–8. 10.1016/j.recqb.2017.04.003

[40] Ko EY Nile SH Guan KV Li H Park SW. Effect of different exposed lights on quercetin and quercetin glucoside content in onion (*Allium cepa* L). Saudi J Biol Sci. (2015) 22:398–403. 10.1016/j.sjbs.2014.11.012 26150744 PMC4486465

[41] Wilawan N Ngamwonglumlert L Devahastin S Chiewchan N. Changes in enzyme activities and amino acids and their relations with phenolic compounds contents in okra treated by LED lights of different colors. Food Bioproc. Tech. (2019) 2:1945–54. 10.1007/s11947-019-02359-y

[42] Liu Y Fang S Yang FW Shang X Fu XX. Light quality affects flavonoid production and related gene expression in *Cyclocarya paliurus*. J Photochem Photobiol B: Biol. (2018) 179:66–73. 10.1016/j.jphotobiol.2018.01.002 29334625

[43] Lee MK Arasu MV Park S Byeon DH Park SU Chung SO. LED lights enhance metabolites and antioxidants in Chinese cabbage and kale. Braz Arch Biol Technol. 59:e16150546. 10.1590/1678-4324-2016150546

[44] Kim K Kook HS Jang YJ Lee WH Kamala-Kannan S Chae JC. The effect of blue-light-emitting diodes on antioxidant properties and resistance to *Botrytis cinerea* in tomato. J Plant Pathol Microb. (2013) 4:9.

[45] Karamać M Koleva L Kancheva VD Amarowicz R. The structure–antioxidant activity relationship of ferulates. Molecules. (2017) 22:527. 10.3390/molecules22040527 28346342 PMC6154093

[46] McAusland L Lim MT Morris DE Smith-Herman L Mohammed U Hayes-Gill BR. Growth spectrum complexity dictates aromatic intensity in coriander (*Coriandrum sativum* L.). Front Plant Sci. (2020) 11:462. 10.3389/fpls.2020.00462 32499791 PMC7242725

