# Supplementary material for: Changes in Functional Compounds, Volatiles, and Antioxidant Properties of Culinary Herb Coriander Leaves (Coriandrum sativum) Stored Under Red and Blue LED Light for Different Storage Times
Source: Front Nutr. 2022 May 12;9:856484. doi: 10.3389/fnut.2022.856484 (PMC9134111; doi:10.3389/fnut.2022.856484)
Supplement: Supplementary file 1 [file Table_1.docx]

Supplementary Table 1. illustrating the respective calibration equations and the LOD, and LOQ values for the standards

| Standard | Regression equation | R^2^ | LOD µg mL^-1^ | LOQ µg mL^-1^ |
| --- | --- | --- | --- | --- |
| Gallic acid | Y=99324x+55626 | 0.9975 | 0.05 | 0.186 |
| Ellagic acid | Y=20110x+9484.9 | 0.9968 | 0.31 | 1.0 |
| Syringic acid | Y=75813x-107617 | 0.9987 | 0.05 | 0.18 |
| Rosmarinic acid | Y=27146x+260505 | 0.9959 | 3.8 | 10.6 |
| Kaempferol | Y=26898x+330818 | 0.9963 | 2.7 | 9.32 |
| Quercetin | Y=12091x-170181 | 0.9964 | 7.7 | 25.8 |
| Protocatechuic acid | Y=19722x-341718 | 0.9983 | 3.2 | 10.9 |
| Ferulic acid | Y=20067x-279209 | 0.9989 | 3.3 | 11.0 |
| p-Coumaric acid | Y=6925.3x-62646 | 0.9989 | 10.25 | 34.17 |
| Caffeic acid | Y=28189x-161653 | 0.9987 | 2.2 | 7.20 |
